# BAF53A drives colorectal cancer development by regulating DUSP5-mediated ERK phosphorylation

**DOI:** 10.1038/s41419-022-05499-w

**Published:** 2022-12-16

**Authors:** Ziqing Yang, Dandan Huang, Manqi Meng, Wencong Wang, Junyan Feng, Lekun Fang, Honglei Chen, Shaomin Zou

**Affiliations:** 1 Guangdong Provincial Key Laboratory of Colorectal and Pelvic Floor Disease, The Sixth Affiliated Hospital, Sun Yat-sen University, 510655 Guangzhou, China; 2 Guangdong Institute of Gastroenterology, 510655 Guangzhou, China; 3 Department of Colorectal Surgery, The Sixth Affiliated Hospital, Sun Yat-sen University, 510655 Guangzhou, China; 4 Gastrointestinal Endoscopy Center, The Eighth Affiliated Hospital, Sun Yat-sen University, 518033 Shenzhen, China

**Keywords:** Cell signalling, Colorectal cancer

## Abstract

BAF53A, an important subunit of the SWI/SNF epigenetic chromatin regulatory complex, has been implicated as the driver of diverse cancers. However, the role of BAF53A in colorectal cancer (CRC) remains poorly understood. Here, we examined the expression of BAF53A in CRC samples and observed that BAF53A was significantly upregulated in CRC tissues compared with paired adjacent normal tissues. In vitro and in vivo studies suggested that ectopic expression of BAF53A promoted colorectal cancer cell proliferation, colony formation, and tumorigenesis, whereas knockdown of BAF53A hindered these cellular functions. DUSP5 (dual-specificity phosphatase 5), an ERK1/2-specific endogenous phosphatase, was expressed at low levels in CRC. We found a negative correlation between BAF53A and DUSP5 expression in a set of CRC samples. Mechanistic studies revealed that P63 was a potential transcription repressor of DUSP5. BAF53A could interact with P63, decreasing the DUSP5 expression level and subsequently promoting ERK1/2 phosphorylation. Thus, our study provides insights into the applicability of the BAF53A-DUSP5-ERK1/2 axis as a potential therapeutic target in CRC.

## Introduction

Colorectal cancer (CRC) is the third and second most common cancer diagnosis and cause of cancer death globally [1, 2]. Surgery remains the most effective treatment for early CRC [3]. Chemotherapy, immunotherapy, and molecular-targeted therapy also play important roles in the prognosis [4]. However, the survival rate is still low because the majority of CRC patients are diagnosed at advanced stages. Hence, it is urgent to uncover the molecular mechanisms involved in CRC progression, which may provide more biomarkers and therapeutic targets.

BAF53A, a member of the ATP-dependent switching (SWI)/sucrose fermentation (SNF) complex, has been implicated as an oncogenic driver, including CRC, hepatocellular carcinoma, glioma, and squamous cell carcinoma [5–9]. Previous studies have shown that BAF53A is involved in diverse cellular processes, including transcriptional regulation and chromatin remodeling [5]. BAF53A could interact with P63, cooperatively controlling a transcriptional program that promotes squamous cell carcinoma cell proliferation and suppresses differentiation [5]. Additionally, it has been shown that BAF53A physically binds to YAP/TAZ and disrupts the interaction between YAP and β-Trcp, which promotes YAP protein degradation in glioma [7]. However, the functional roles of BAF53A in CRC remain to be explored.

Mitogen-activated protein kinase (MAPK), one of the major oncogenic intracellular signaling pathways in CRC, plays a key role in tumor cell proliferation, invasion, and metastasis [10]. The well-known members of the MAPK family include ERK, JNK, and p38 [11]. Abnormalities in MAPKs cause multiple diseases [12]. Of note, the largest group of proteins that specifically regulate MAPK activity in mammalian cells is the dual-specificity phosphatase (DUSP) family [13]. All DUSPs contain a common phosphatase domain, dephosphorylating both threonine/serine and tyrosine residues of their substrates [11]. Different members of the DUSP family display distinct substrate specificities for diverse MAPKs and subcellular localization [14]. DUSP5 is an ERK-specific endogenous phosphatase that localizes to the nucleus [15]. Under endoplasmic reticulum stress, DUSP5 upregulation leads to hepatocyte death [16]. Depletion of DUSP5 results in paclitaxel resistance and poor prognosis in breast cancer [17]. ARAP1-AS1 promotes cervical cancer progression by negatively modulating DUSP5 [18]. Additionally, DUSP5 also acts as a key target of linc01503 in gastric cancer [19]. Therefore, unveiling the precise mechanisms that govern DUSP5 expression is expected to lead to the exploration of alternative strategies for cancer treatment.

Here, we characterize that BAF53A promotes CRC progression. DUSP5, the phosphatase of ERK pathways, is observably upregulated after BAF53A depletion. Moreover, BAF53A activates P63-mediated DUSP5 promoter activity by interacting with P63, resulting in ERK1/2 phosphorylation. We also reveal the clinical correlation between BAF53A and DUSP5, which provides a therapeutic rationale for targeting the BAF53A-DUSP5 axis in CRC.

## Results

### BAF53A expression is upregulated in colorectal cancer and promotes CRC cell proliferation

**Fig. 1:**
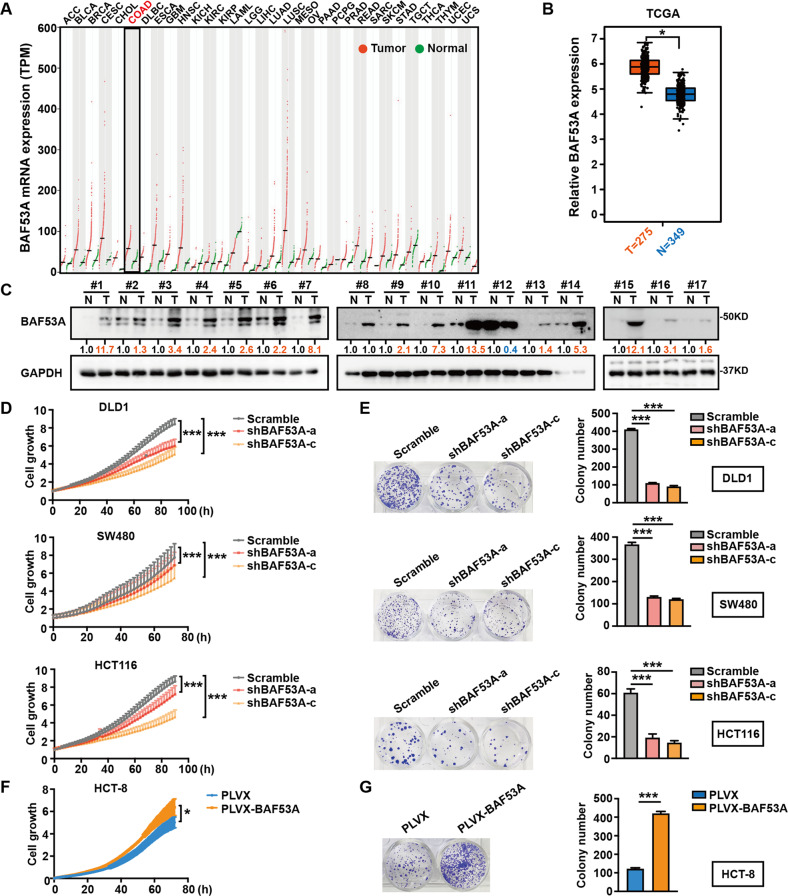
BAF53A is overexpressed in CRC and required for the proliferation of CRC cells. **A** BAF53A expression across various non-tumorous and tumorous tissues (unit, transcript per million TPM). Each dot represents information from one patient. COAD: colon adenocarcinoma. **B** Relative expression of BAF53A in normal (N) and CRC tissue samples (T) from the TCGA, *P < 0.05. **C** BAF53A expression in 17 paired samples of CRC tumors (T) and corresponding adjacent normal tissues (N). **D**, **E** BAF53A knockdown inhibited the proliferation of CRC cells. Incucyte and colony formation assays were performed to examine the effect of BAF53A knockdown on cell viability, ***P < 0.001. **F**, **G** Overexpression of BAF53A promoted the proliferation of HCT-8 cells. Incucyte and colony formation assay were performed to examine the effect of BAF53A overexpression on cell viability, *P < 0.05, ***P < 0.001.

To explore the roles of BAF53A in CRC, we examined GEPIA and The Cancer Genome Atlas (TCGA) and found that the BAF53A expression level was higher in CRC tissues than in normal tissues (Fig. 1A, B). In addition, analysis of the expression of BAF53A in 17 pairs of matched fresh frozen primary CRC tissues and adjacent normal mucosa revealed that CRC exhibited high BAF53A levels (Fig. 1C). Previous study revealed that BAF53A promoted invasion, metastasis, and epithelial–mesenchymal transition of colon cancer [8]. To further investigate the biological role of BAF53A in CRC, we knocked down BAF53A in the CRC cell lines DLD1, SW480, and HCT116 using shRNAs. Incucyte assays showed that knockdown of BAF53A significantly slowed the growth of CRC cells (Fig. 1D). Consistent with this finding, depletion of BAF53A in CRC cells also caused significant inhibition of colony formation (Fig. 1E). Conversely, BAF53A overexpression significantly promoted HCT-8 cell growth (Fig. 1F). Enhanced cell proliferation by BAF53A upregulation was also observed in colony formation assays (Fig. 1G). Collectively, BAF53A is highly expressed in CRC, and its positive impacts on cell proliferation contribute to its oncogenic role.

### BAF53A drives CRC cell growth by attenuating DUSP5

**Fig. 2:**
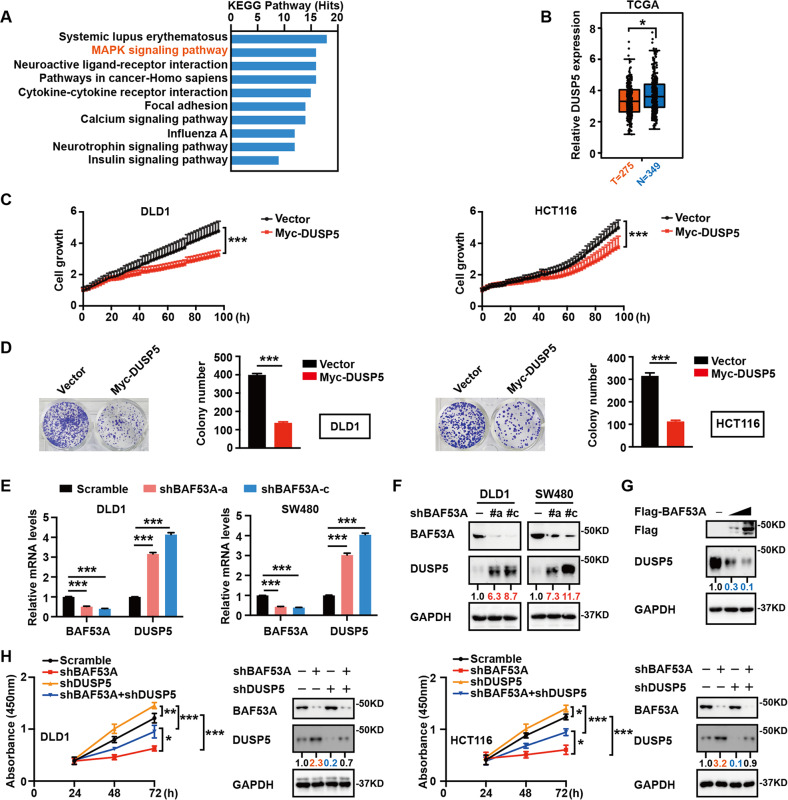
BAF53A drives CRC cell growth by attenuating DUSP5. **A** Top 10 enriched KEGG pathways of differentially expressed BAF53A target genes in CRC. **B** Relative expression of DUSP5 in normal and CRC tissue samples from the TCGA, *P < 0.05. **C**, **D** Incucyte and colony formation assays showed inhibited viability after DUSP5 was overexpressed in DLD1 and HCT116 cells compared to control, ***P < 0.001. **E**, **F** DUSP5 mRNA and protein levels were analyzed after BAF53A depletion. The data are presented as the means ± SD, ***P < 0.001. **G** Overexpression of BAF53A decreased the steady-state protein levels of DUSP5. **H** CCK8 assays showed that knockdown of BAF53A could suppress CRC cell proliferation, while depletion of DUSP5 could rescue it. *P < 0.05, **P < 0.01, ***P < 0.001. Western blot showed the knockdown efficiency of the shRNAs.

To elucidate the molecular mechanisms of BAF53A in promoting CRC cell growth, we performed RNA sequencing and figured out that MAPK signaling is one of the top ten KEGG pathways manipulated by BAF53A (Fig. 2A). Dual-specificity phosphatase 5 (DUSP5), an inhibitory component of the MAPK pathway, targets and dephosphorylates ERK1 and ERK2 [20]. TCGA analysis showed that DUSP5 expression was lower in tumor tissues than in matched normal tissues (Fig. 2B). Additionally, DUSP5 overexpression reduced DLD1 and HCT116 cell proliferation and colony formation (Fig. 2C, D). These findings indicate that DUSP5 acts as a tumor suppressor in CRC.

We next characterized the relevance of BAF53A and DUSP5. Both the mRNA and protein expression of DUSP5 were significantly increased after BAF53A depletion (Fig. 2E, F). In contrast, BAF53A overexpression reduced DUSP5 expression levels (Fig. 2G). Furthermore, suppression of DUSP5 abrogated BAF53A depletion-induced cell growth inhibition (Fig. 2H), indicating that BAF53A-induced proliferation of CRC cells was dependent on DUSP5.

### BAF53A activates ERK phosphorylation by suppressing DUSP5

**Fig. 3:**
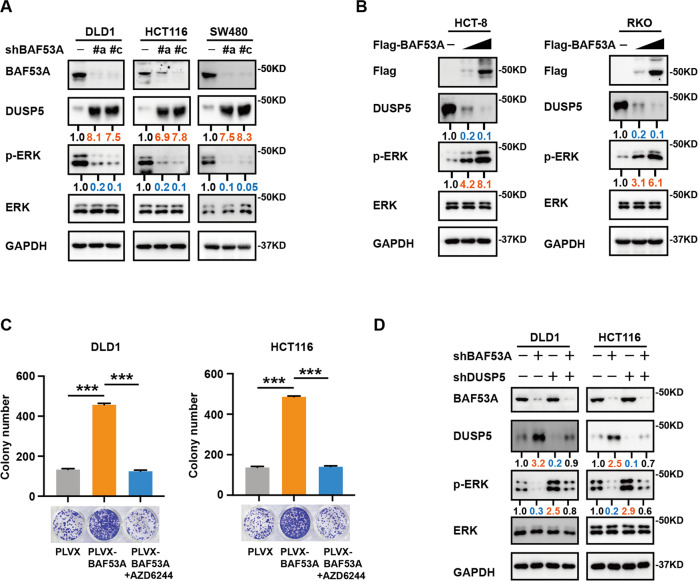
BAF53A actives ERK phosphorylation by suppressing DUSP5. **A** Western blot analysis of BAF53A, DUSP5, p-ERK, ERK, and GAPDH from lysates of CRC cells infected with the indicated shRNAs. **B** Western blot showed that the DUSP5 protein level was decreased while p-ERK expression level was upregulated when BAF53A was overexpressed. **C** AZD6244 treatment (0.1 μM) abrogated BAF53A-induced DLD1 and HCT116 cell colony formation, ***P < 0.001. **D** Cell lysates from DLD1 and HCT116 cells infected with shBAF53A or/and shDUSP5 were immunoblotted with the indicated antibodies.

Since DUSP5 is a specific phosphatase of ERK1/2 [13], we then explored whether ERK1/2 signaling was regulated by BAF53A. Indeed, inhibition of BAF53A resulted in decreased phosphorylation of ERK1/2 (Fig. 3A). Constitutive overexpression of BAF53A promoted phosphorylation of ERK1/2 in CRC cells (Fig. 3B). AZD6244, a highly selective MEK inhibitor, inactivates ERK1/2 phosphorylation. To further test whether the BAF53A-ERK axis can impact CRC cell growth, we treated cells with AZD6244 after overexpression of BAF53A. As shown in Fig. 3C, AZD6244 treatment abrogated BAF53A-induced CRC cell colony formation. Moreover, BAF53A depletion decreased ERK1/2 phosphorylation, which could be rescued by knocking down DUSP5 (Fig. 3D), suggesting that BAF53A was able to modulate DUSP5-induced ERK1/2 phosphorylation.

### Identification of P63 as a transcription repressor of DUSP5

**Fig. 4:**
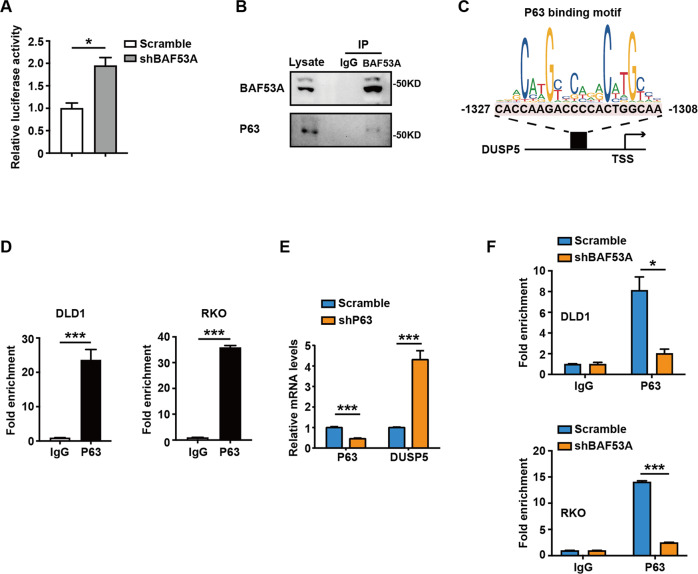
BAF53A interacts with P63 to suppress DUSP5 promoter activity. **A** Loss of BAF53A could increase DUSP5 promoter activity. The data are presented as the means ± SD, *P < 0.05. **B** The interaction between BAF53A and P63 was determined by a co-immunoprecipitation (co-IP) assay. **C** Predicted binding site of P63 in DUSP5 promoter. P63 binding motif was obtained from the website: http://jaspar.genereg.net/. **D** Chromatin was precipitated from DLD1 and RKO cells with antibodies against P63, or IgG, and analyzed by qPCR (mean ± SD). ***P < 0.001. **E** Relative mRNA levels of P63 and DUSP5 in the scramble and shP63 DLD1 cells. ***P < 0.001. **F** Chromatins were precipitated from DLD1 and RKO cells infected with BAF53A shRNA or scramble, using anti-P63 or anti-IgG antibodies, followed by RT-qPCR. The data are presented as the means ± SD, *P < 0.05, ***P < 0.001.

We showed that loss of BAF53A increased DUSP5 promoter activity (Fig. 4A), which indicated that BAF53A regulated DUSP5 at a transcriptional level. It has been well documented that BAF53A and P63 coordinately regulate key genes [5]. Therefore, we proposed that P63, a crucial transcription factor, might act as a mediator of BAF53A and DUSP5. First, the association of BAF53A and P63 was explored by immunoprecipitation assay in DLD1 cells. As expected, BAF53A interacted with P63 (Fig. 4B). Then we performed the dual luciferase reporter assays and found that loss of p63 could also increase DUSP5 promoter activity (supplementary Fig. 1A). In addition, chromatin immunoprecipitation followed by qPCR (ChIP–qPCR) assays supported that P63 directly binds to the promoter regions of DUSP5 (Fig. 4C, D). When we knocked down P63, the mRNA level of DUSP5 was increased (Fig. 4E), indicating that P63 could function as a transcription repressor of DUSP5. Furthermore, the binding of P63 to the promoter of DUSP5 was markedly impaired in BAF53A-depleted cells (Fig. 4F). BRG1 is the catalytic subunit of the SWI/SNF chromatin-remodeling complex. We found that BRG1 could bind to the promoter regions of DUSP5 (supplementary Fig. 1B), suggesting that BAF53A promotes p63-mediated transcriptional repression of DUSP5 through chromatin remodeling by the BAF complex.

### Roles of BAF53A in promoting CRC growth in vivo

**Fig. 5:**
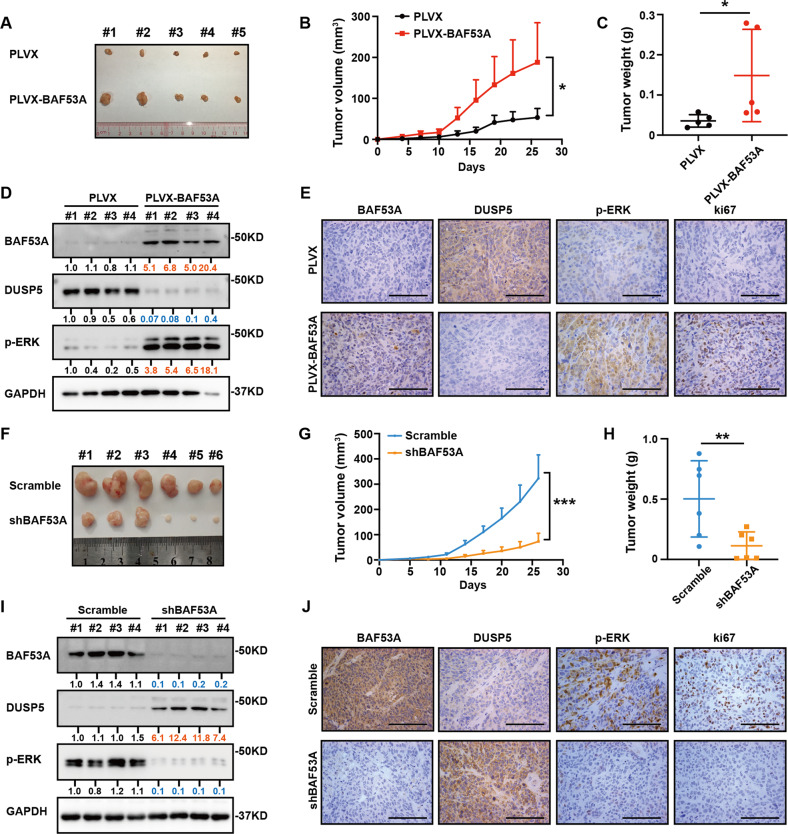
BAF53A drives endogenous CRC tumor progression. **A** HCT116 cells were stably infected with the indicated lentivirus and injected s.c. into BALB/c-nude mice. Four weeks after injection, xenografts were removed. Representative images of xenografts were shown. **B**, **C** Tumor volume (**B**) and tumor weight (**C**) were determined. *P < 0.05. **D** Western blot analysis of BAF53A, DUSP5, and p-ERK protein levels in xenografts. **E** Immunohistochemical analysis of the expression of BAF53A, DUSP5, p-ERK, and ki67 in different groups of xenografts. Scale bar: 100 µm. **F** DLD1 cells infected with Scramble or shBAF53A were implanted subcutaneously into BALB/c-nude mice to form xenografts. After four weeks, the xenografts were harvested. Representative images of xenografts were shown. **G**, **H** Tumor volume (**G**) and tumor weight (**H**) were determined. **P < 0.01, ***P < 0.001. **I** Western blot analysis of BAF53A, DUSP5, and p-ERK protein levels in xenografts. **J** Immunohistochemical analysis of the expression of BAF53A, DUSP5, p-ERK, and ki67 in xenografts. Scale bar: 100 µm.

We next established xenograft models using HCT116 cells stably infected with PLVX or PLVX-BAF53A. Both the volume and the weight of tumors obtained from the BAF53A-overexpression group were clearly increased compared with those from the control group (Fig. 5A–C). Moreover, western blot and IHC staining revealed that tumors formed by cells infected with BAF53A showed sharply decreased expression of DUSP5 and significantly increased expression of p-ERK and Ki67 (Fig. 5D, E). In contrast, BAF53A downregulation significantly decreased the tumor size and tumor weight relative to the control group (Fig. 5F–H). In addition, tumors formed by DLD1 cells infected with shBAF53A displayed DUSP5 upregulation and lower expression of p-ERK and Ki67 (Fig. 5I, J). Together, these data confirmed that BAF53A could promote CRC tumorigenesis.

### The BAF53A–DUSP5 axis correlates with the clinical outcomes of colorectal cancer patients

**Fig. 6:**
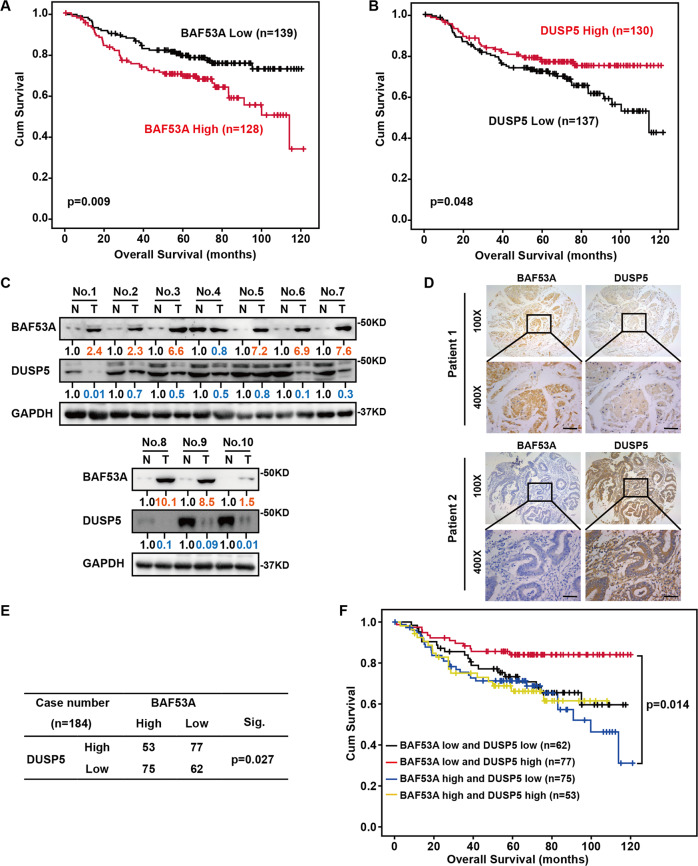
Clinical significance of BAF53A–DUSP5 axis in CRC. **A**, **B** Kaplan–Meier estimates of survival time of patients in cohort (*n* = 267) by different BAF53A (**A**) or DUSP5 (**B**) expression levels in tumors. **C** Western blot analysis showed the expression of BAF53A and DUSP5 in ten CRC tumor tissues compared with matched adjacent normal tissues. **D** Representative images showing negative correlations between protein levels of BAF53A and DUSP5 in CRC specimens. Scale bar, 50 μm. **E** Percentages of samples showing the relationship among BAF53A and DUSP5. *P* = 0.027 by chi-square test. **F** Kaplan–Meier curves of the overall survival of patients with different expression levels of BAF53A and DUSP5. *P* = 0.014 by log-rank test.

To determine the clinical relevance between BAF53A and DUSP5, we examined their expression profiles in the cohort of CRC cancer tissues. Clinically, patients with high BAF53A or low DUSP5 expression levels were correlated with worse prognosis (Fig. 6A, B). Additionally, a negative correlation between the protein level of BAF53A and DUSP5 was demonstrated in CRC than in paired normal tissues (Fig. 6C). We further assessed the clinical relevance of the above-described findings by using a tissue microarray with 267 CRC tissue specimens (Table 1). The expression of BAF53A was negatively correlated with DUSP5 by immunohistochemistry (IHC) analysis (Fig. 6D, E). Additionally, based on BAF53A and DUSP5 expression levels, the samples were classified into four groups: High BAF53A and Low DUSP5, High BAF53A and High DUSP5, Low BAF53A and Low DUSP5, and Low BAF53A and High DUSP5 expression. Kaplan–Meier analysis suggested that patients with high BAF53A and low DUSP5 expression tended to have the poorest overall survival compared with the other groups (Fig. 6F). These data imply that the BAF53A–DUSP5 axis plays a role in human cancer development.

**Table 1:** Correlation between the expression of BAF53A–DUSP5 axis and clinicopathological features of colorectal cancer patients.

Variable	BAF53A expression			DUSP5 expression		
	Low	High	*P* value^a^	Low	High	*P* value^a^
Gender			0.179			0.714
Male	77	60		72	65	
Female	62	68		65	65	
Age			0.540			0.222
<59 years	68	57		59	66	
≥59 years	71	71		78	64	
Histological grade			0.882			0.839
G1	11	9		11	9	
G2	105	100		106	99	
G3	23	19		20	22	
pT status			0.068			0.035
T1	2	7		6	3	
T2	17	18		25	10	
T3	116	103		105	114	
T4	4	0		1	3	
pN status			0.799			0.525
N0	87	83		90	80	
N1	52	45		47	50	
pM status			0.178			0.699
M0	127	110		123	114	
M1	12	18		14	16	
Clinical stage			0.397			0.210
I	15	17		22	10	
II	63	56		58	61	
III	49	37		43	43	
IV	12	18		14	16	

Note: All data are no. of patients (%).

^a^*P* values were calculated in SPSS16.0 using a Pearson Chi-Square Test (Fisher’s Exact Test was used when >20% of cells have an expected count <5). *P* values <0.05 were considered to indicate statistical significance.

## Discussion

In mammalian cells, there are two major types of SWI/SNF complexes, including the canonical BAF (BRG1-associated factors) complex, which incorporates ARID1A or ARID1B subunits, and the PBAF (polybromo-associated BAF) complex, which contains ARID2, PBRM1 and BRD7 subunits [21]. Many studies have elucidated that SWI/SNF chromatin-remodeling complexes play important roles in the pathogenesis and progression of multiple cancers [8, 9, 22–24]. It has been reported that the SWI/SNF subunit BAF53A acts as an EMT activator in colon cancer [8]. Here, we identified the roles of BAF53A in CRC cell growth and tumorigenesis. Knockdown of BAF53A hindered CRC cell growth both in vivo and in vitro. Nevertheless, mammalian SWI/SNF complexes consist of at least 8-12 subunits, and further investigations can be carried out to explore whether the BAF53A function depends on other subunits.

To characterize the function of BAF53A in CRC, we performed RNA sequencing and identified the MAPK pathway as the top downstream signaling pathway of BAF53A. ERK1/2 belongs to the MAPK family. The RAS-RAF-MEK-ERK pathway is altered in nearly 40% of human cancers and plays key roles in cell proliferation, differentiation, and apoptosis [25, 26]. Dual-specific phosphatase is a unique group of proteins that counteract MAPKs [11], of which DUSP5 is the phosphatase targeting and anchoring ERK1/2. Through ectopic expression and knockdown studies, we found that BAF53A-induced ERK1/2 phosphorylation and CRC progression could be partially reversed by DUSP5, indicating the important role of DUSP5 in mediating BAF53A-induced CRC progression.

P63 and p73 were identified as p53 homologs in the late 1990s. P63 can be expressed in multiple isoforms (ΔNP63α/β/γ/δ/ε and TaP63α/β/γ/δ/ε). The ΔN forms lack a transactivation domain. Notably, it has been documented that p53 can directly bind to the promoter region of the DUSP5 gene [27]. Previous study provided compelling evidence that BAF53A is physically associated with p63 and function together in a common pathway to drive a refractory cancer phenotype in HNSCC [5]. Similarly, we showed that BAF53A and p63 co-regulated DUSP5, and this transcriptional regulation was direct, as these two factors were co-localized to the transcription start site (TSS) of DUSP5. BAF53A, as a member of the SWI/SNF chromatin-remodeling complex, could bind to p63 and promote its chromatin accessibility, thereby repressing DUSP5 transcriptionally.

In vivo models and human tumors further support the relevance of the BAF53A-DUSP5 axis in CRC progression. Here, we demonstrate that BAF53A overexpression is sufficient to drive tumorigenesis associated with DUSP5 inhibition and ERK1/2 activation. In contrast, loss of BAF53A blocks tumor growth and induces DUSP5 expression in vivo. Accordingly, BAF53A expression is strongly inversely correlated with DUSP5 in human CRC samples. Furthermore, high BAF53A and/or low DUSP5 are significant determinants of poor overall survival in CRC patients. Taken together, we provide new ideas for nascent therapeutic approaches for CRC.

In summary, our research uncovered the oncogenic role of BAF53A in CRC. Mechanistically, BAF53A interacts with P63, a transcription repressor of DUSP5, thereby decreasing the DUSP5 expression level and subsequently inducing ERK1/2 phosphorylation. Our research highlights a greater understanding of the BAF53A-mediated molecular network and provides potential therapeutic targets for CRC.

## Materials and methods

### Cell culture and transfection

The human colon cancer cell lines DLD1, SW480, HCT116, HCT-8, and RKO were obtained from American Type Culture Collection (ATCC) and cultured in RMPI-1640 (Roswell Park Memorial Institute) or Minimum Essential Medium (MEM) medium supplemented with 10% fetal bovine serum (FBS). All cell lines were tested and authenticated by assessing the morphology, proliferation rate, genetic markers, and checking for mycoplasma-negative.

The cDNAs for human BAF53A were cloned by RT-PCR and subcloned into pcDNA3.1 (+) vector or PLVX vector. All plasmids were validated by DNA sequencing. Transient transfections of plasmids into cell lines using Polyethylenimine HCl MAX, Linear, Mw 40,000 (Polysciences Inc., 24765-1) followed the manufacturer’s protocol.

### Quantitative PCR (qPCR)

Total RNA was extracted using TRizol (Invitrogen) and then reverse transcribed into cDNA according to the manufacturer’s protocol (TOYOBO, FSQ-301). Quantitative PCR (qPCR) was performed on LightCycler480 PCR system (Roche) using SYBR Green Premix (biotool, #B21203). The primer sequences were listed as follows: *BAF53A* F: TGGAGGCCATTTCACCTCTAA; *BAF53A* R: TCTTTGCTCTAGTATTCCACGGT;

*DUSP5* F: TGTCGTCCTCACCTCGCTA;

*DUSP5* R: GGGCTCTCTCACTCTCAATCTTC;

*P63* F: GGACCAGCAGATTCAGAACGG;

*P63* R: AGGACACGTCGAAACTGTGC;

*GAPDH* F: GGAGCGAGATCCCTCCAAAAT;

*GAPDH* R: GGCTGTTGTCATACTTCTCATGG.

### RNA sequence and gene set enrichment analysis (GSEA)

DLD1 cells were infected twice with scramble shRNA and shBAF53A lentivirus for 48 h. RNA samples from DLD1 cells were isolated using the Trizol reagent (Invitrogen) and then sent for sequence (Shanghai Biotechnology Corporation). To analyze the difference between scramble shRNA groups and shBAF53A groups, we performed the Gene set enrichment analysis (GSEA) by the JAVA program (https://www.gsea-msigdb.org/gsea/index.jsp) using KEGG v7.0. symbols gene set collection.

### Immunoprecipitation and immunoblot assays

Cells were collected and resuspended by lysis buffer (50 mM Tris-Cl, pH 7.5, 0.1% NP-40, 0.1% Triton-100, 150 mM NaCl, 0.1 M EDTA, a cocktail of phosphate and proteinase inhibitors) and incubated with BAF53A antibody or the control IgG antibody, rotation at 4 °C overnight. Protein A/G agarose beads (50 μL, Santa Cruz, SC-2001) were then added to each sample. After 3 h, the beads were washed and followed by immunoblotting.

For immunoblot assays, proteins were separated by sodium dodecyl sulfate (SDS)-polyacrylamide gel electrophoresis. After transfer and blocking, the membrane sections were incubated with indicated antibodies, and horseradish peroxidase-linked secondary antibody (1:5000, Thermo, 31430). Anti-BAF53A (1:1000, Abcam, ab3882), anti-DUSP5 (1:1000, Abcam, ab200708), anti-pERK (1:2000, Cell Signaling Technology, 4370), anti-ERK (1:1000, Cell Signaling Technology, 4695S), anti-P63 (1:1000, Cell Signaling Technology, 13109S), anti-GAPDH (1:1000, Proteintech, 60004-1-Ig), anti-Flag (1:1000, Cell Signaling Technology, 8146S) antibodies were used.

### Cell proliferation assay

For the Incucyte assay, cells were seeded in 6-well plates and then put into the machines, which could analyze cell growth by long-time real-time dynamic living cell imaging.

For the CCK8 assay, infected cells were seeded into 96-well plates and incubated overnight. CCK-8 solution (APExBIO) was then added to each well every 24 h and finally measured the samples at 450 nm by a microplate reader.

### Colony formation assay

Cells were seeded into 6-well plates (800–1000 cells per well) and then cultured for 10–14 days. when visible colonies formed, fixed the cells with 4% paraformaldehyde (PFA) and stained them with crystal violet. Colony numbers were finally counted by image J. All the data were repeated at least three times.

### Luciferase reporter assay

Infected cells were co-transfected with Firefly luciferase reporters and indicated plasmids using Polyethylenimine HCl MAX, Linear, Mw 40,000 (Polysciences Inc., 24765-1). Twenty-four hours later, Dual-Luciferase Reporter Assay Kit (Promega, E1960) was used for detection, and Renilla activity was used to normalize.

### Chromatin immunoprecipitation–quantitative PCR (ChIP-qPCR)

Chromatin immunoprecipitation was performed following the fast ChIP protocol with minor modifications. Briefly, cells were collected and crosslinked with 1.42% formaldehyde, then disrupted by ultrasonication. The sonicated chromatins were incubated with indicated antibodies at 4 °C overnight with rotation. Protein A-agarose beads (sc2003, Santa Cruz) were added into the mixtures and rotated for 2 h at 4 °C. Next, the immunoprecipitates and input DNA samples were treated with Chelex 100 resin (BioRad) and incubated at 100 °C for 10 min to reverse crosslinking. After proteinase K treatment, enriched DNA fragments were subjected to RT-PCR analysis. IgG was used as the negative control. Primers of the DUSP5 promoter are shown:

F: -GAAAGAAGGCCCCCGCAT; R: 5’-GTCACAGATGCATCCACCCAA.

### Immunohistochemistry staining

For immunohistochemical staining, paraffin-embedded tumor specimens were cut into slices (Servicebio). After the slides are baked, conventional dewaxing with xylene solution and dehydration with different gradients of alcohol is carried out. Antigen repair, block (Origene), and then sections were put in a 4 °C refrigerator for incubation of primary antibody overnight. Following incubation with biotinylated goat anti-rabbit or anti-mouse IgG at room temperature for 30 min, diaminobenzidine and hematoxylin were used for visualizing the staining.

### In vivo xenograft experiments

Four-week-old female BALB/c nude mice were randomly and blindly divided into two groups (*n* = 5 per group). HCT116 cells stably infected with PLVX or PLVX-BAF53A were inoculated subcutaneously into mice. The tumor volumes were measured and calculated as length × width^2^ × 0.5. Four weeks later, the tumors were removed, photographed, and weighed. Besides, DLD1 cells were infected with BAF53A-shRNA and scramble. Stably infected cells (1 × 10^6^) were subcutaneously injected in the lower rear flank of 4-week-old female BALB/c nude mice (*n* = 6 per group). On day 28 after implantation, tumors were harvested and weighed. Finally, tumors were subjected to protein and IHC analysis.

### Patients and tissue samples

For tissue microarray analysis (TMA), 267 paraffin-embedded samples were collected from the First Affiliated Hospital of Sun Yat-sen University with the patients’ written informed consent and approval from the study center’s Institutional Review Board. The immunostained slides were then scanned by Aperio Versa (Leica Biosystems), which could capture digital images. To define the cut-off point, the receiver operating characteristic curve was used.

### Statistical analyses

Statistical analyses were performed using SPSS software version 22.0. One-way analysis of variance and Student’s *t* test were used to determine differences between groups. The overall survival (OS) rates were analyzed by Kaplan–Meier survival analysis with the log-rank test. Chi-square test was used to evaluate the association between BAF53A and DUSP5 staining intensities. A value of *P* < 0.05 is considered statistically significant. All data were shown as mean ± SEM or mean ± SD.

## Supplementary information

Supplementary Fig. 1

aj-checklist

Supplemental material (WB)

## Data Availability

All data generated or analyzed during this study are available from the corresponding author on reasonable request. The RNA array result is deposited in Gene Expression Omnibus (GEO) under the accession number GSE218008.
