# Supplementary material for: BAF53A drives colorectal cancer development by regulating DUSP5-mediated ERK phosphorylation
Source: Cell Death Dis. 2022 Dec 16;13(12):1049. doi: 10.1038/s41419-022-05499-w (PMC9758165; doi:10.1038/s41419-022-05499-w)
Supplement: Supplementary file 1 — Supplementary Fig. 1 [file 41419_2022_5499_MOESM1_ESM.pdf]

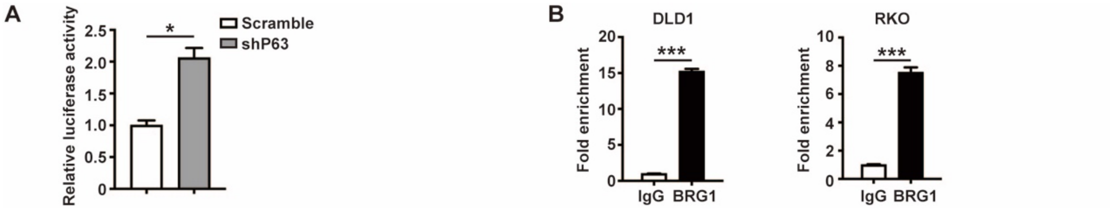

Supplementary Fig. 1 BAF53A promotes p63-mediated transcriptional repression of DUSP5 through chromatin remodeling by the BAF complex. **A** P63 knockdown increased DUSP5 promoter activity. *P<0.05. **B** Chromatin was precipitated from DLD1 and RKO cells with antibodies against BRG1, or IgG, and analyzed by qPCR (mean ± SD). ***P<0.001.
