# Supplementary material for: BAF53A drives colorectal cancer development by regulating DUSP5-mediated ERK phosphorylation
Source: Cell Death Dis. 2022 Dec 16;13(12):1049. doi: 10.1038/s41419-022-05499-w (PMC9758165; doi:10.1038/s41419-022-05499-w)

Fig. 1C (in paper)

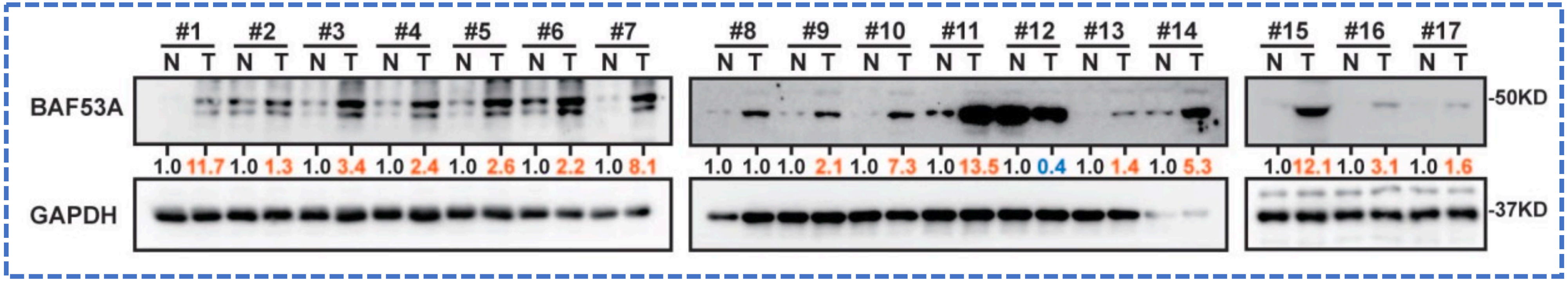

Raw data

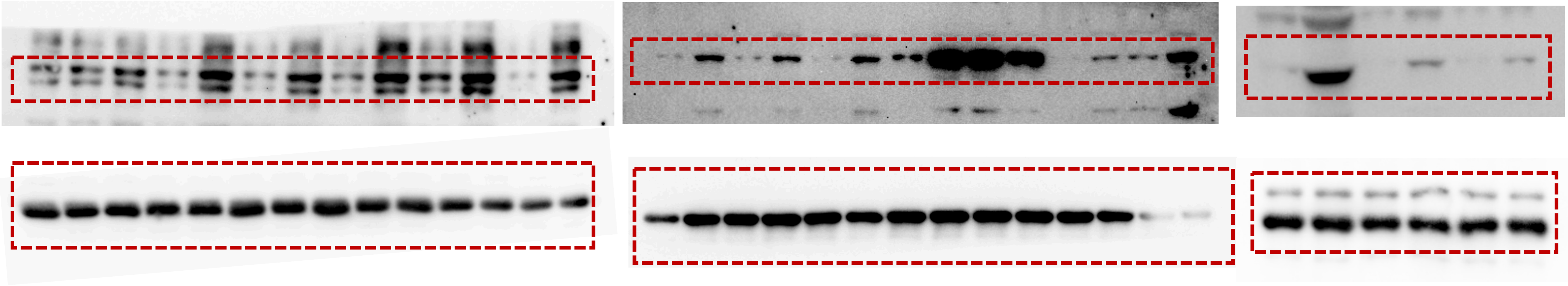

Fig. 2F (in paper)

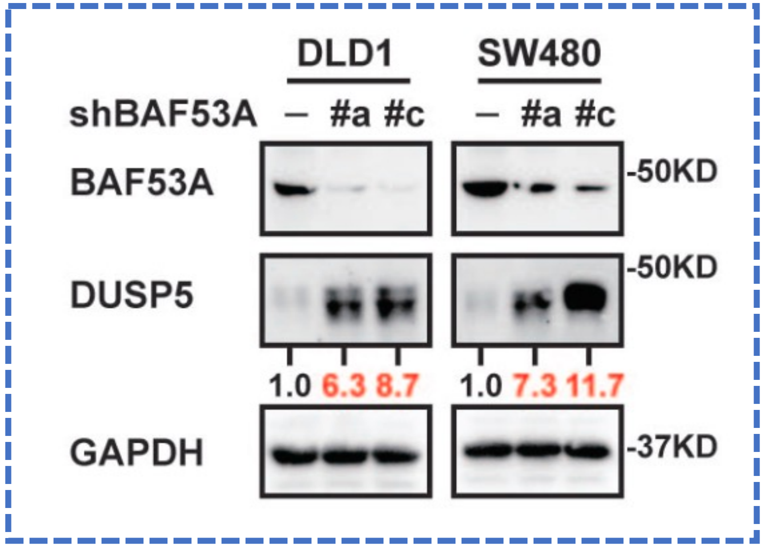

Fig. 2G (in paper)

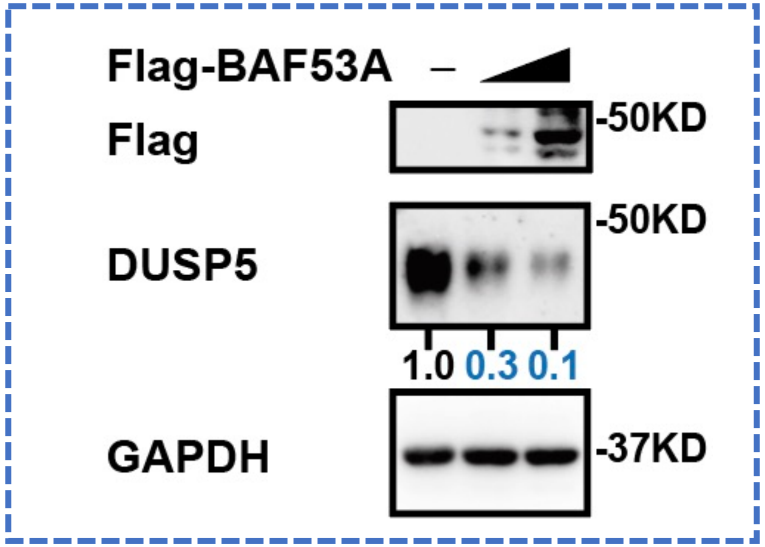

Raw data

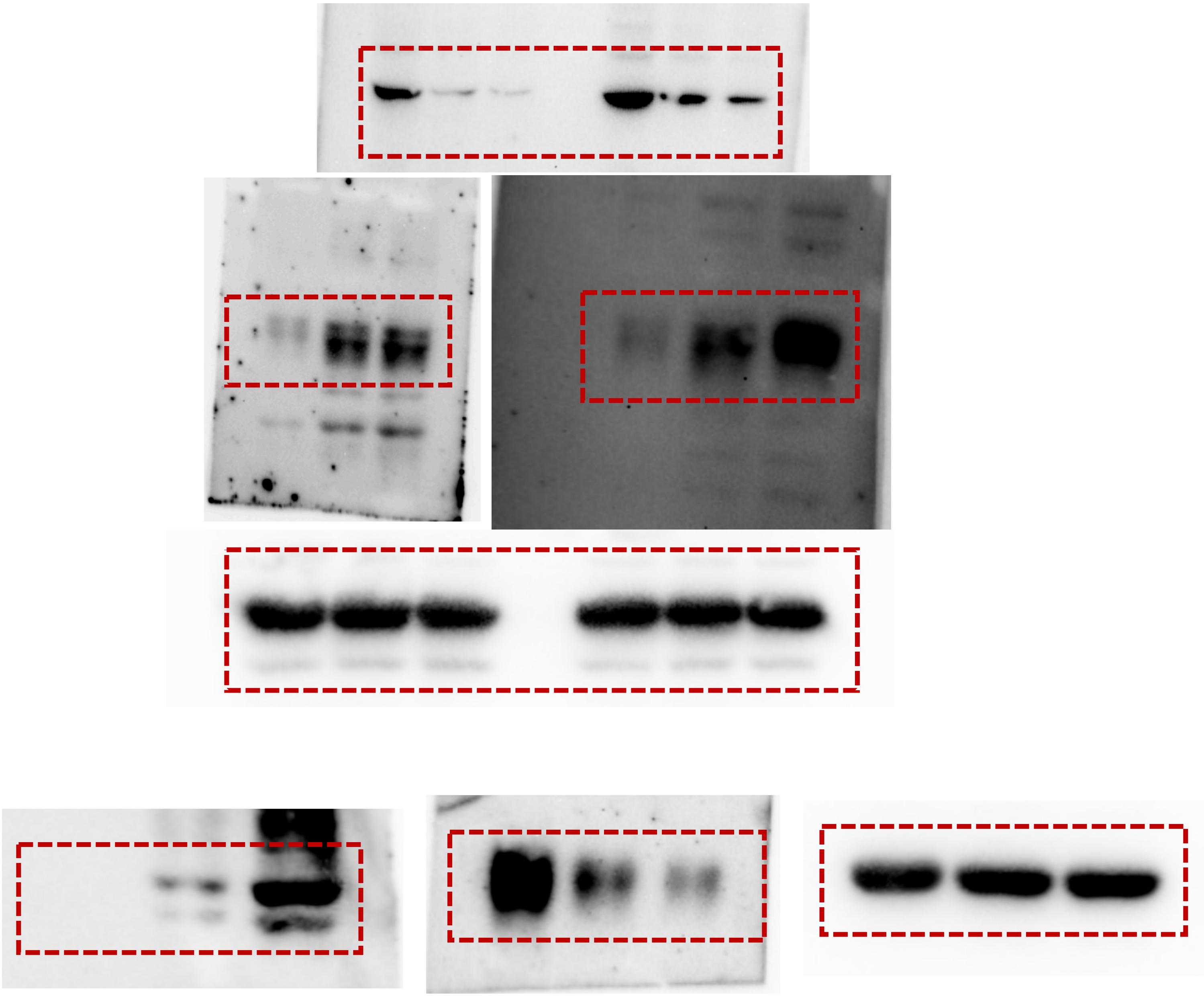

Fig. 2H (in paper)

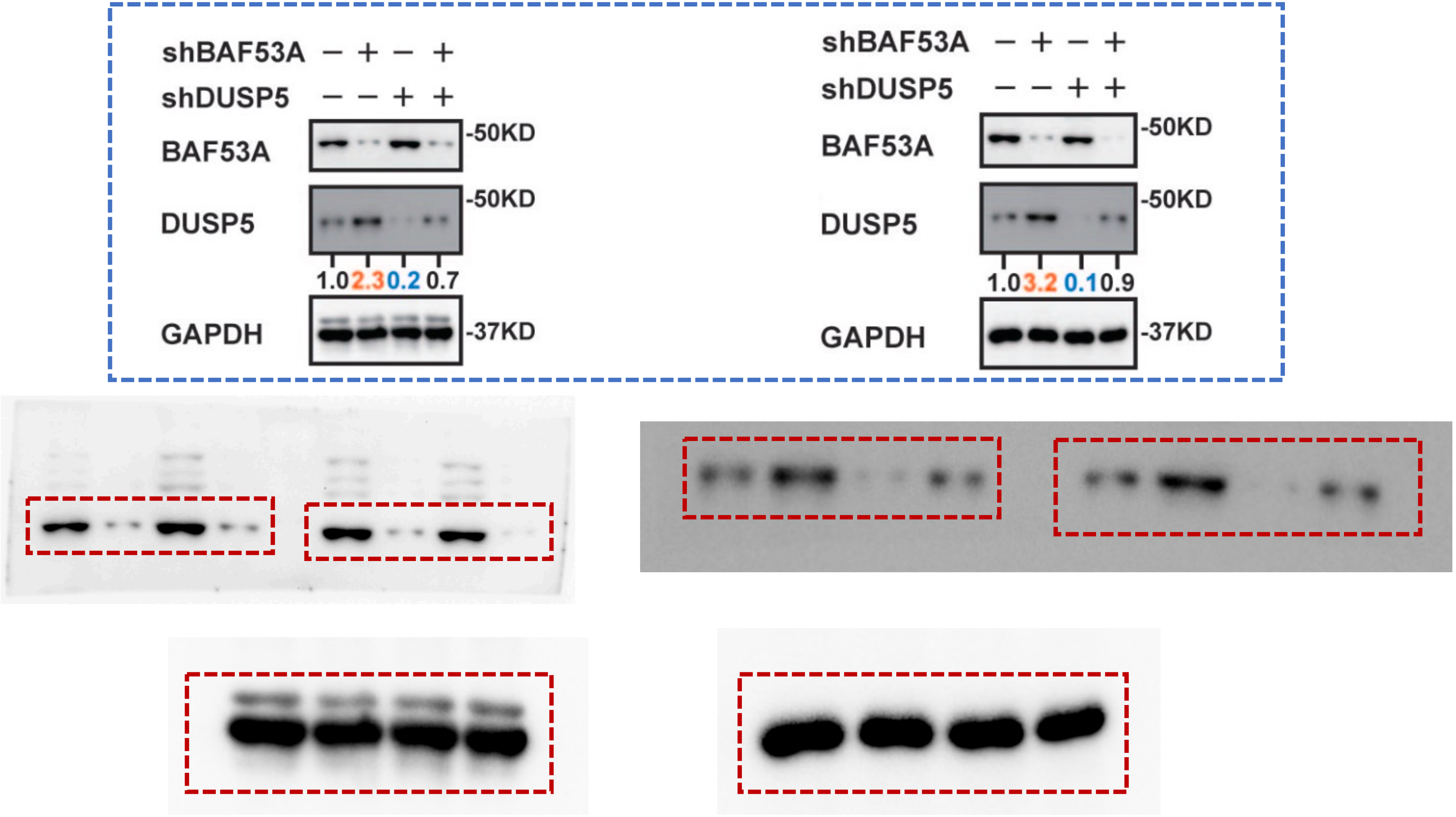

Fig. 3A (in paper)

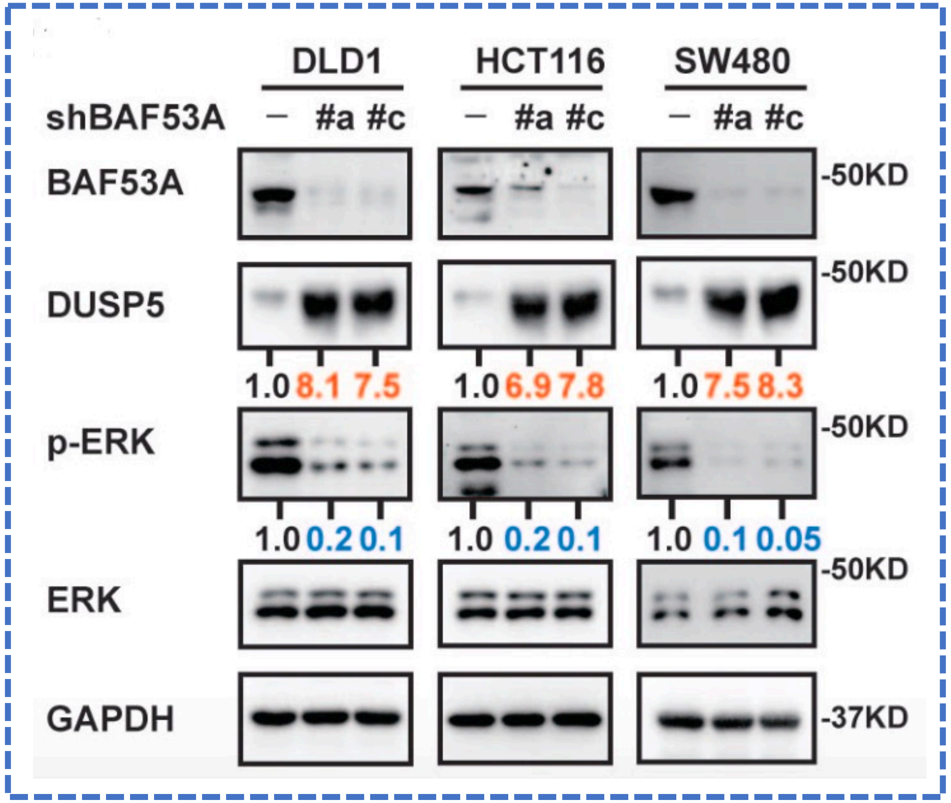

Raw data

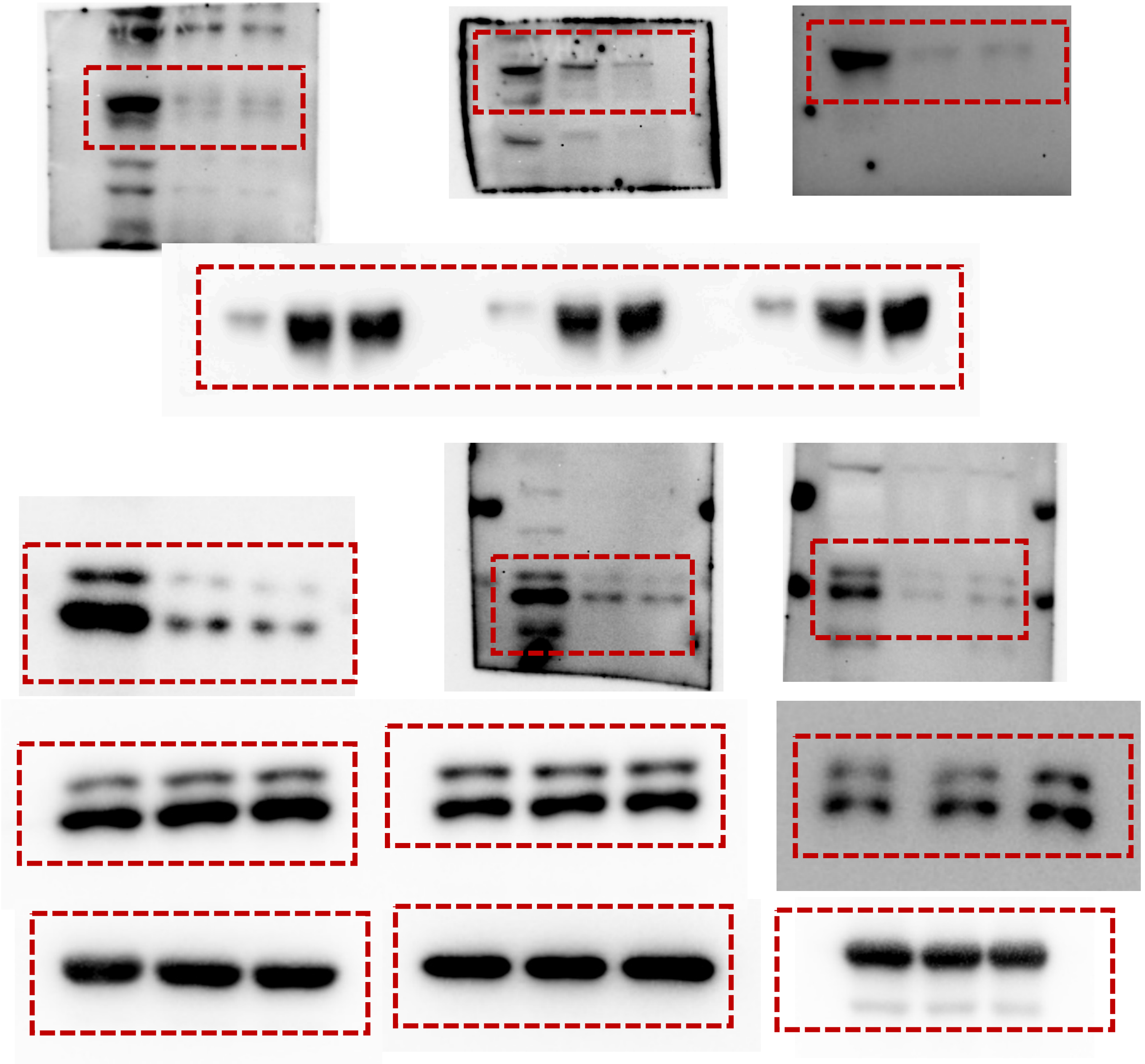

Fig. 3B (in paper)

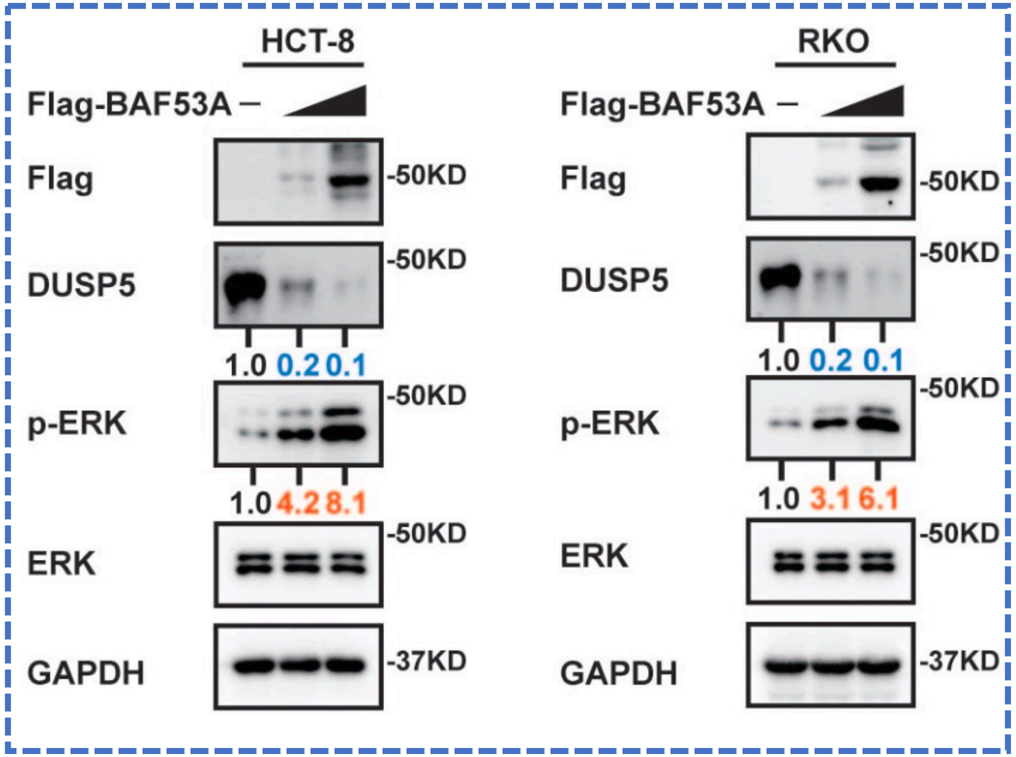

Raw data

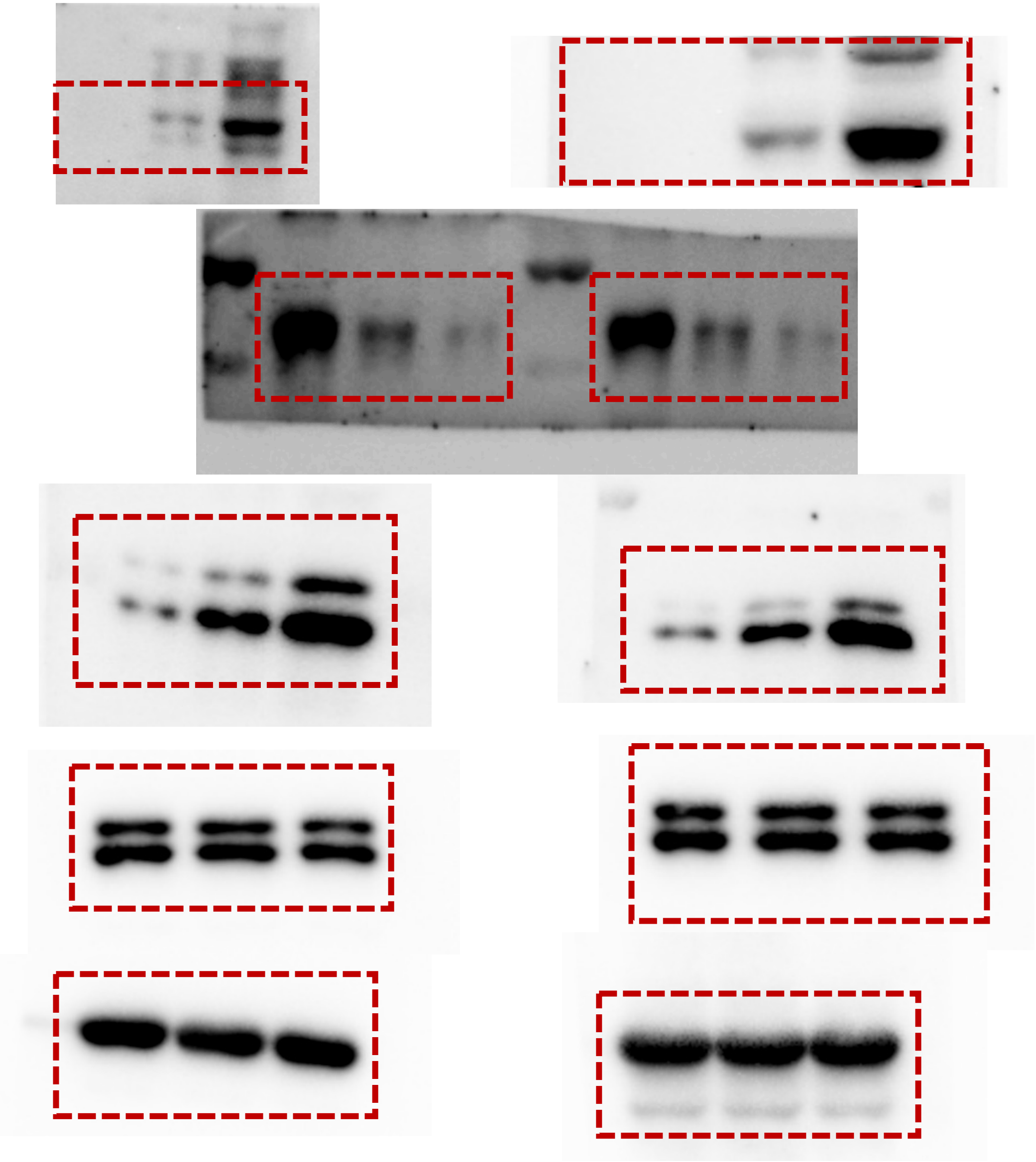

Raw data

Fig. 3D (in paper)

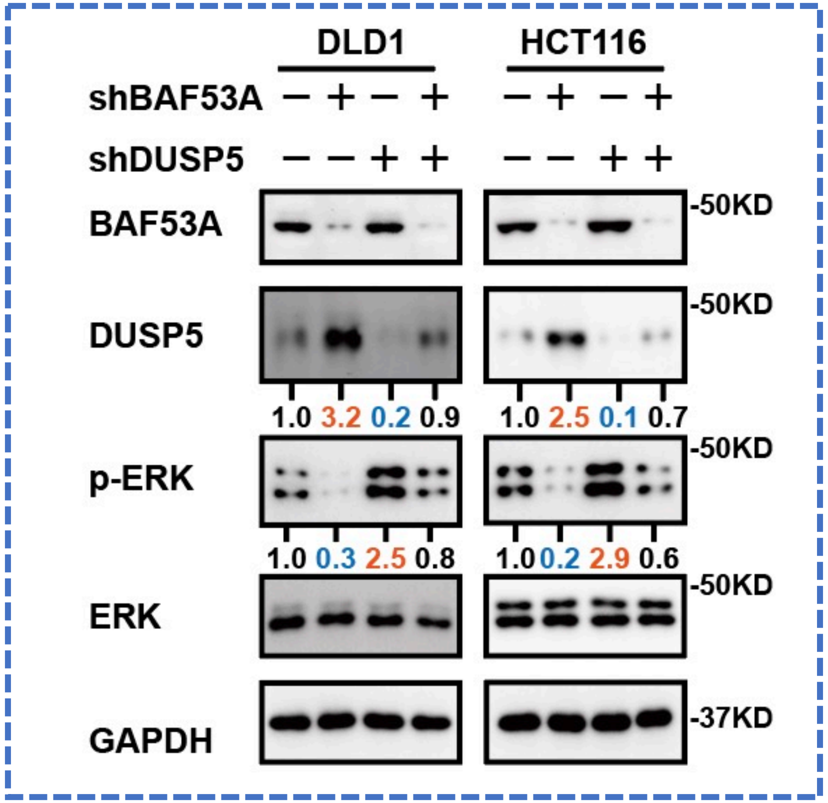

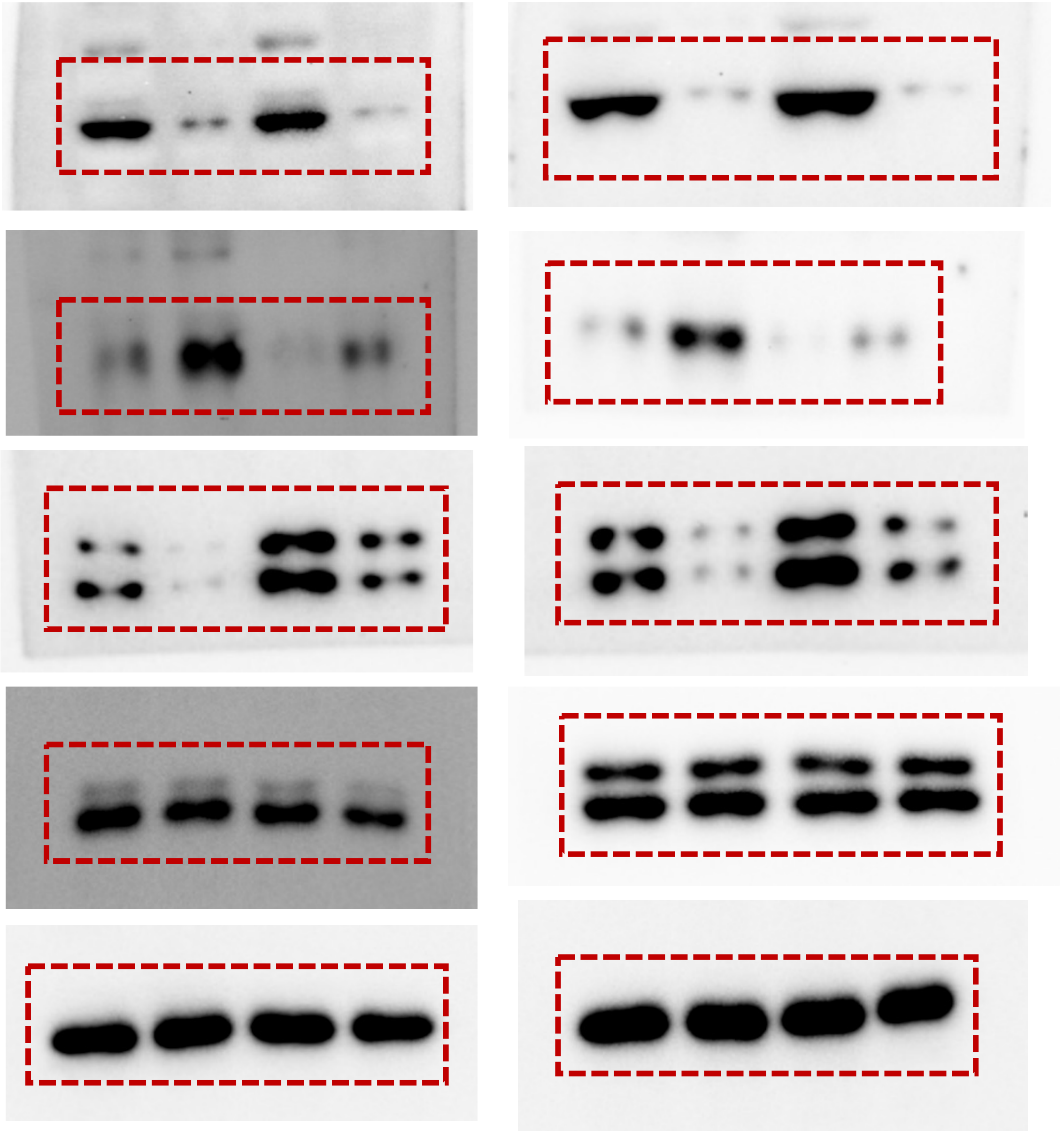

Fig. 4B (in paper)

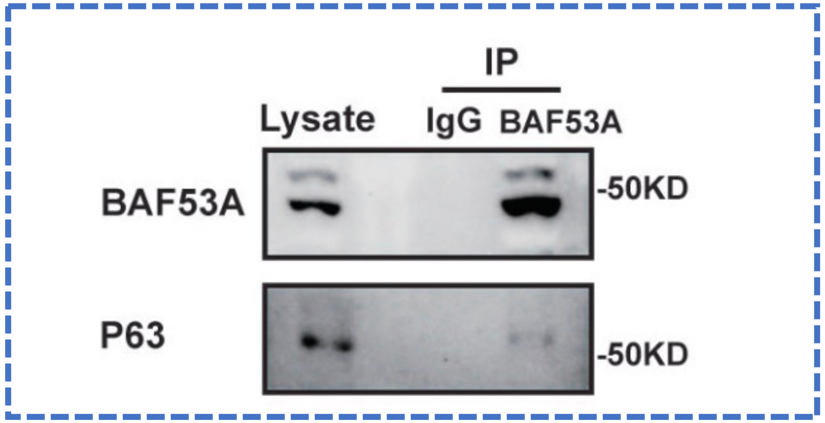

Raw data

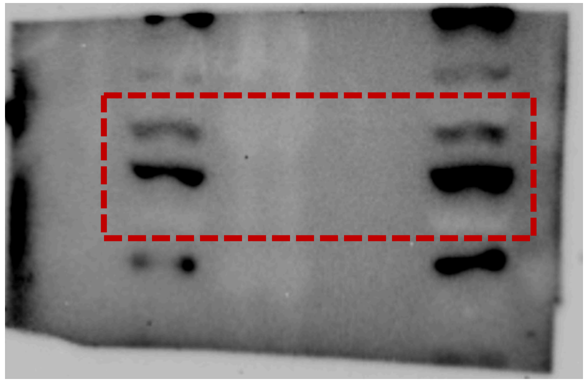

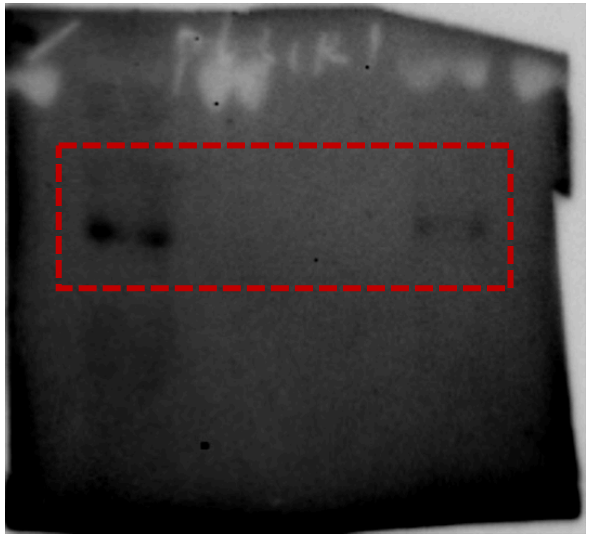

Fig. 5D (in paper)

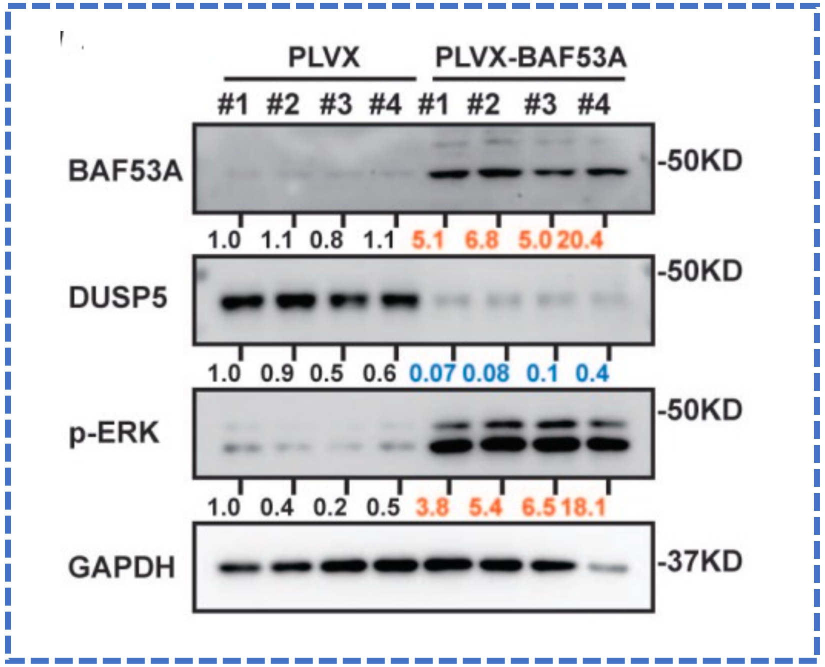

Raw data

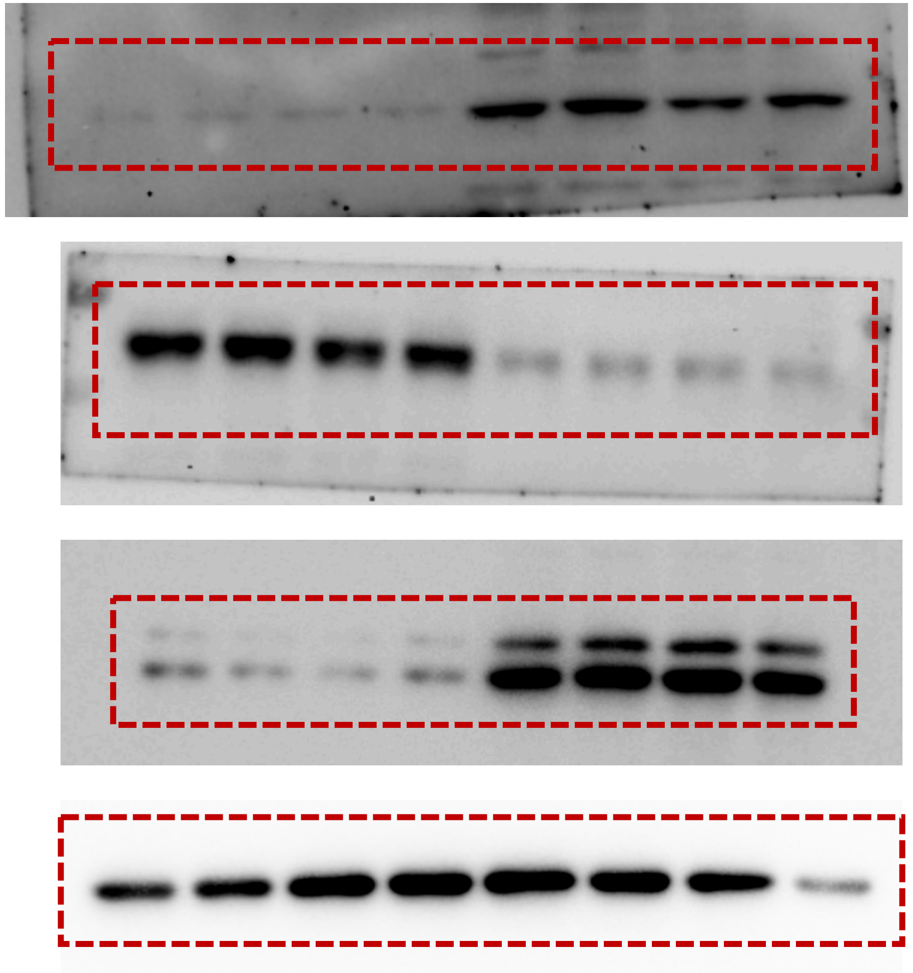

Fig. 5I (in paper)

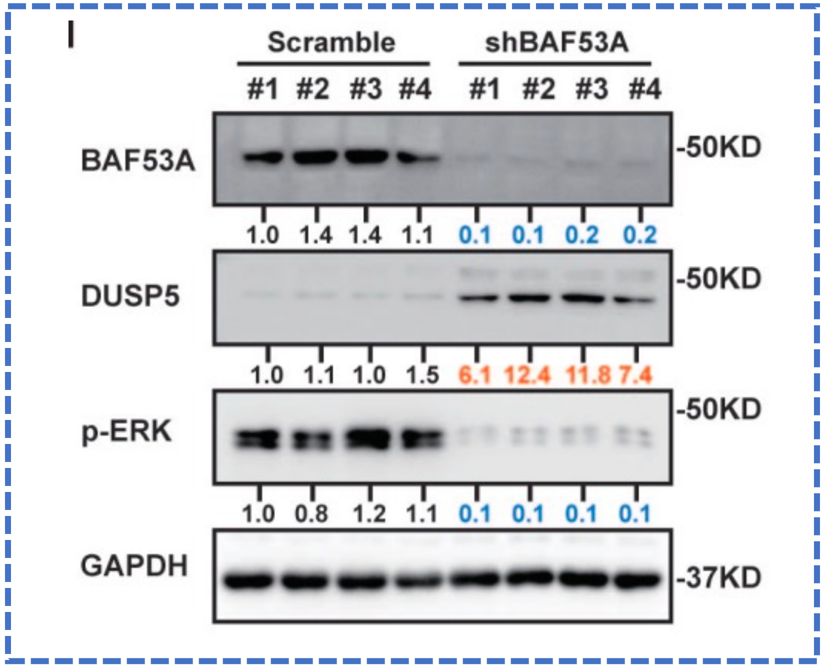

Raw data

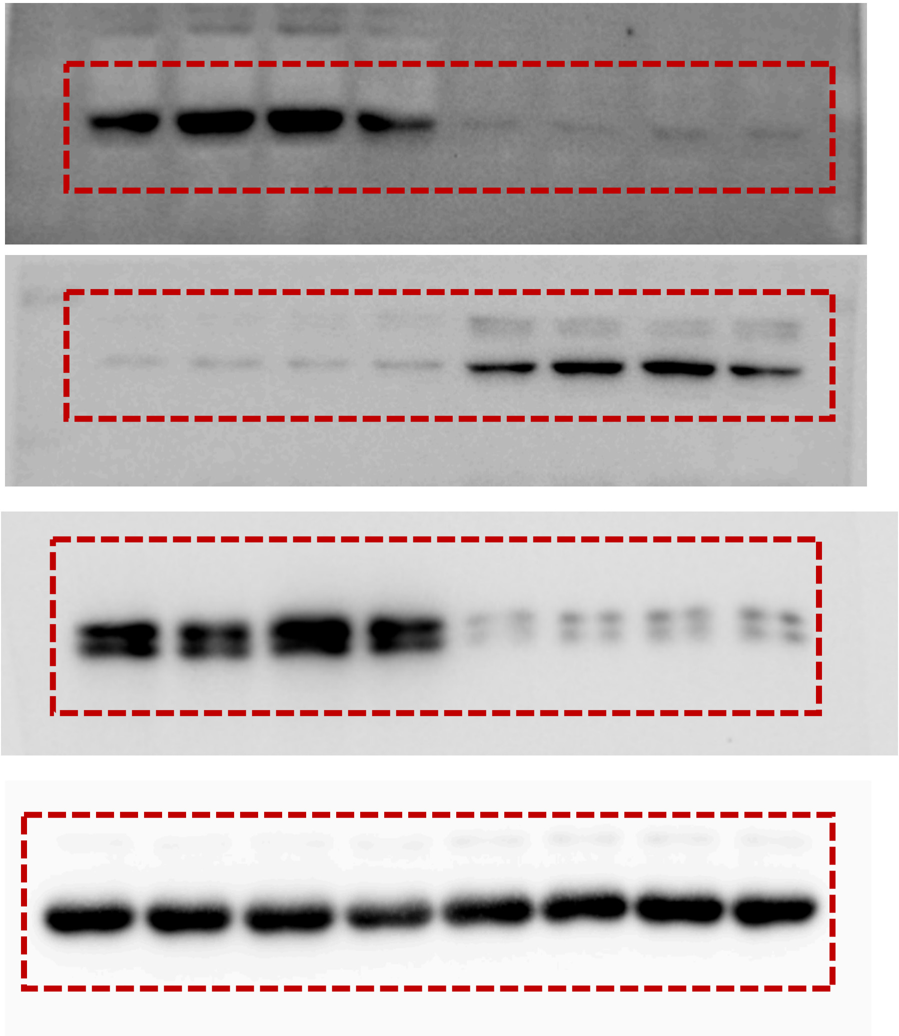

Fig. 6C (in paper)

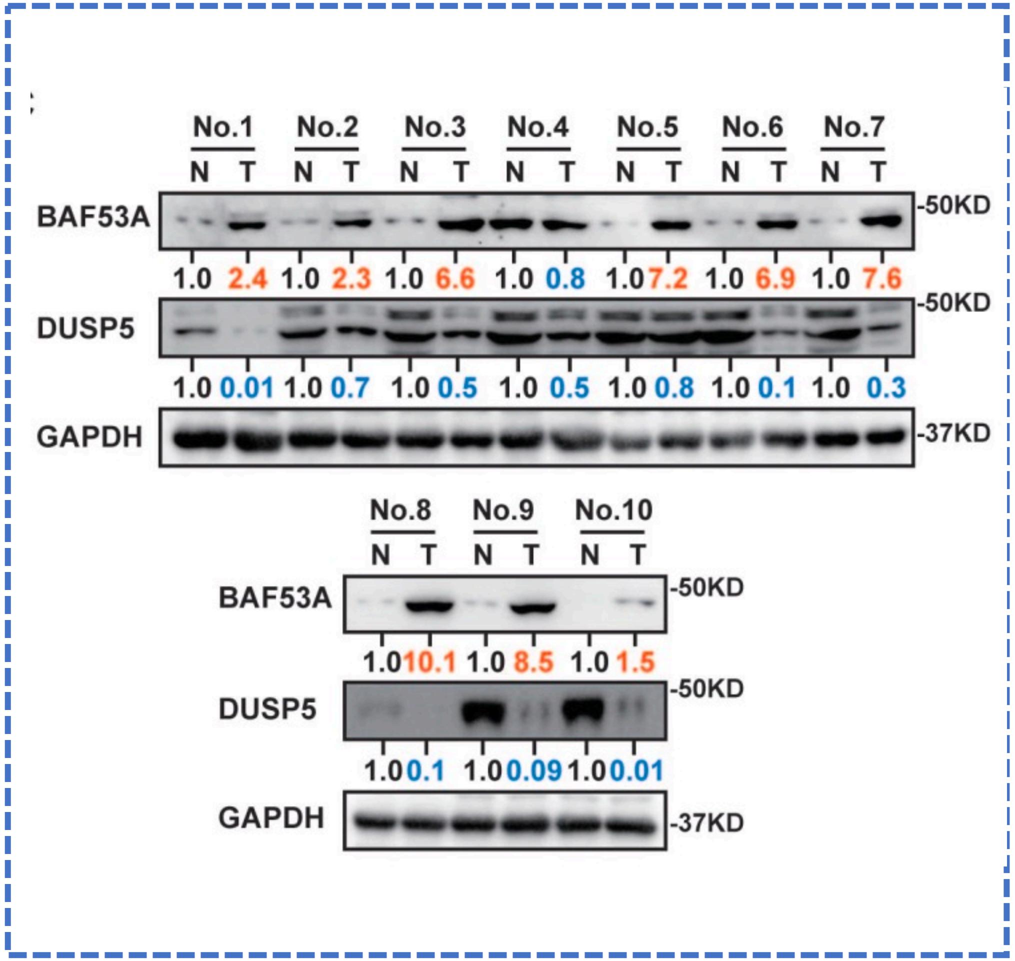

Raw data

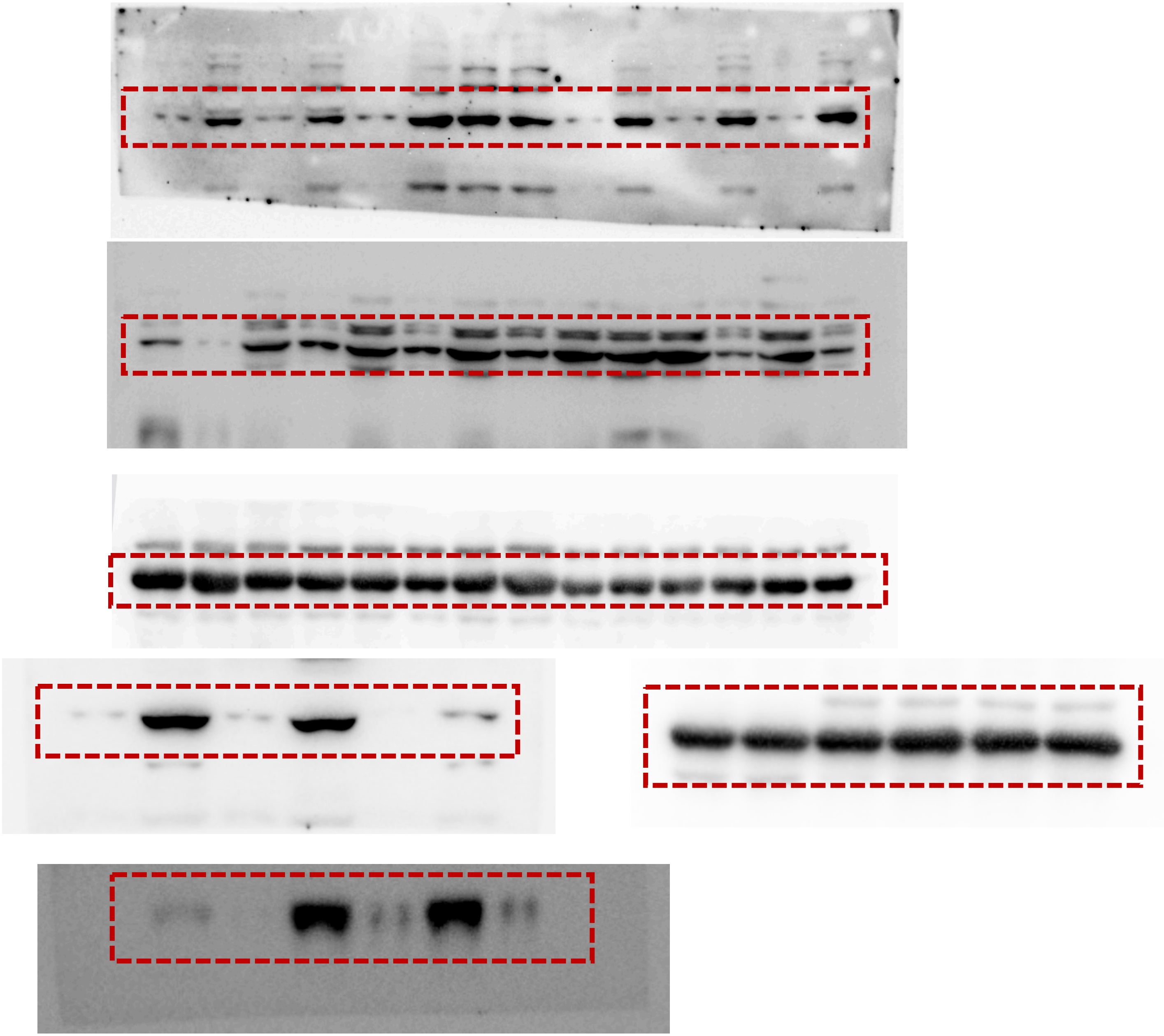

Supplement: Supplementary file 3 — Supplemental material (WB) [file 41419_2022_5499_MOESM3_ESM.pdf]
